# Bacterial Co- or Superinfection in Patients Treated in Intensive Care Unit with COVID-19- and Influenza-Associated Pneumonia

**DOI:** 10.3390/pathogens12070927

**Published:** 2023-07-10

**Authors:** Jochen Johannes Schoettler, Stany Sandrio, Christoph Boesing, Lena Bauer, Thomas Miethke, Manfred Thiel, Joerg Krebs

**Affiliations:** 1 Department of Anaesthesiology and Critical Care Medicine, University Medical Centre Mannheim, Medical Faculty Mannheim of the University of Heidelberg, Theodor-Kutzer-Ufer 1-3, 68165 Mannheim, Germany; stany.sandrio@umm.de (S.S.); christoph.boesing@umm.de (C.B.); lenabauer35@web.de (L.B.); manfred.thiel@medma.uni-heidelberg.de (M.T.); joerg.krebs@umm.de (J.K.); 2 Mannheim Institute for Innate Immunoscience (MI3), Medical Faculty Mannheim, University of Heidelberg, Ludolf-Krehl-Str. 13-17, 68167 Mannheim, Germany; thomas.miethke@umm.de; 3 Institute for Medical Microbiology and Hygiene, University Medical Centre Mannheim, Medical Faculty Mannheim of the University of Heidelberg, Theodor-Kutzer-Ufer 1-3, 68165 Mannheim, Germany

**Keywords:** COVID-19, SARS-CoV-2, influenza, bacterial co- or superinfection, pneumonia, acute respiratory distress syndrome

## Abstract

Viral pneumonia is frequently complicated by bacterial co- or superinfection (c/s) with adverse effects on patients’ outcomes. However, the incidence of c/s and its impact on the outcomes of patients might be dependent on the type of viral pneumonia. We performed a retrospective observational study in patients with confirmed COVID-19 pneumonia (CP) or influenza pneumonia (IP) from 01/2009 to 04/2022, investigating the incidence of c/s using a competing risk model and its impact on mortality in these patients in a tertiary referral center using multivariate logistic regressions. Co-infection was defined as pulmonary pathogenic bacteria confirmed in tracheal aspirate or bronchoalveolar lavage within 48 h after hospitalization. Superinfection was defined as pulmonary pathogenic bacteria detected in tracheal aspirate or bronchoalveolar lavage 48 h after hospitalization. We examined 114 patients with CP and 76 patients with IP. Pulmonary bacterial co-infection was detected in 15 (13.2%), and superinfection was detected in 50 (43.9%) of CP patients. A total of 5 (6.6%) co-infections (*p* = 0.2269) and 28 (36.8%) superinfections (*p* = 0.3687) were detected in IP patients. The overall incidence of c/s did not differ between CP and IP patients, and c/s was not an independent predictor for mortality in a study cohort with a high disease severity. We found a significantly higher probability of superinfection for patients with CP compared to patients with IP (*p* = 0.0017).

## 1. Introduction

Pneumonia caused by influenza or severe acute respiratory syndrome coronavirus 2 (SARS-CoV-2) is associated with relevant morbidity and mortality [1]. In these patients, bacterial co- or superinfection (c/s) is a common complication that might prolong the necessity of mechanical ventilation and the time in the intensive care unit (ICU) and might also be an independent risk factor of mortality [2,3]. For hospitalized coronavirus disease 19 (COVID-19) patients, Lansbury et al. [4] showed in their meta-analysis that the average proportion of bacterial co-infection was 7%, increasing to 14% for ICU patients and was associated with increased mortality. The most common pathogens in this cohort were *Mycoplasma pneumoniae*, *Pseudomonas aeruginosa*, *Haemophilus influenzae*, and *Klebsiella pneumoniae*. Other studies showed an incidence of c/s in patients with COVID-19 pneumonia (CP) between 3 and 6% [5,6,7]. Rozencwajg et al. [8] recently showed that bacterial co-infection in influenza-associated acute respiratory distress syndrome (ARDS) could be detected in 30% of all patients managed with extracorporeal membrane oxygenation (ECMO) and was associated with increased mortality. Various reports identify *Staphylococcus aureus*, *Streptococcus pneumoniae*, *Haemophilus influenzae*, and *Aspergillus* spp. As the most frequently detected pathogens causing co-infection in patients with influenza pneumonia (IP) [8,9,10]. Therefore, the incidence and significance of c/s in patients with CP or IP remains of clinical relevance, especially with respect to patients’ outcomes. In this retrospective mono-center study, we evaluate the incidence of bacterial c/s and its relevance to mortality in patients with CP or IP in a tertiary referral center. The secondary objectives were to evaluate the associated bacteria and the specific characteristics of the patients with c/s.

## 2. Materials and Methods

### 2.1. Study Design

For this retrospective observational, monocenter case-control study, the need for informed consent was waived by the local ethics committee (Medizinische Ethikkommission II, University Medical Centre Mannheim, Medical Faculty Mannheim of the University of Heidelberg) (registration number 2018-862R-MA). This study was registered at the German Clinical Trials Register (DRKS) (registration number: DRKS00029634) and conducted in the 24-bed ICU of a tertiary referral center (Department of Anaesthesiology and Critical Care Medicine, University Medical Centre Mannheim, Medical Faculty Mannheim of the University of Heidelberg). Data were prospectively collected and retrospectively analyzed. The inclusion period lasted from 01/2009 to 04/2022, with an average of 1869 patients per year in the ICU.

### 2.2. Collection of Data

Data were collected through the Philips IntelliVue Clinical Information Portfolio (ICIP) and Philips Intelli Space Critical Care and Anesthesia (ICCA) System. All patients with diagnosed CP or IP confirmed by polymerase chain reaction (PCR) performed on bronchoalveolar lavage, tracheal aspirate, or nasal/throat swab were included in this study. Pneumonia and the severity of the gas exchange impairment due to pneumonia were diagnosed and categorized according to current guidelines [11,12,13].

Subsequently, patients were differentiated by c/s according to the following definitions.

Bacterial co-infection: detection of one or more pathogenic bacterial species in a respiratory sample within 48 h after hospitalization.

Bacterial superinfection: detection of one or more pathogenic bacterial species in a respiratory sample 48 h after hospitalization.

These definitions used for c/s in our study followed recent publications [4,5,6,8,14,15,16].

Additionally, in this study, each microbiological isolate was reviewed by an experienced senior ICU consultant to determine the clinical significance; bacteria identified but not warranting specific therapy were defined as commensal and nonsignificant.

If multiple pathogenic bacteria were detected in a patient, this was considered as one c/s. However, the different bacteria were also considered in the analysis.

### 2.3. Statistical Analysis

Statistical analysis was performed with JMP^®^ Version 15 from SAS, SAS Version 9.4 (SAS, Cary, NC, USA), and Prism Version 9.5.1 (GraphPad Software, San Diego, CA, USA). Metric data are presented as mean ± standard deviation, and categorical data as absolute frequency (percentage). *p*-values were calculated using the *t*-test, Mann–Whitney *U* test, or Fisher’s exact test, as appropriate. For the analysis of superinfection incidence, we considered mortality as a competing risk and patients discharged from the ICU as censored when fitting a Fine-Gray sub-distribution hazard model with the disease group (CP or IP) as the independent variable. The corresponding cause-specific hazard ratios for superinfection and mortality were also derived with conventional proportional hazards models. Such models were also used to assess the association between superinfection and mortality in CP and IP patients. In addition to these univariable models, models including the SOFA score on ICU admission were also analyzed. Univariable analysis of factors associated with mortality was performed using logistic regression. Multivariable analysis was used to further evaluate factors significantly associated with mortality in the univariable analysis. A *p*-value ≤ 0.05 was regarded as statistically significant.

## 3. Results

### 3.1. Study Population

All patients treated in the ICU of a tertiary referral center from 01/2009 to 04/2022 were included in the analysis. We identified 114 patients with CP as well as 76 patients with IP. In total, 104 patients with CP and 74 patients with IP fulfilled ARDS criteria according to the Berlin definition [13]. Patients’ anthropometric characteristics are summarized in Table 1. A total of 89 (78.1%) patients with CP and 75 (98.7%) patients with IP were mechanically ventilated. Patients with CP were significantly older than patients with IP (61 ± 16 vs. 49 ± 13 years, *p* < 0.0001). In contrast, patients with IP had a significantly higher sequential organ failure assessment (SOFA) score (13.4 ± 2.3 vs. 9.4 ± 3.9, *p* < 0.0001). Duration of mechanical ventilation in these patients was longer (19.5 ± 14.8 vs. 16.5 ± 16.3 days, *p* = 0.0377), and they were more likely to require ECMO therapy (48.7% vs. 24.6%, *p* < 0.001).

**Figure 1:**
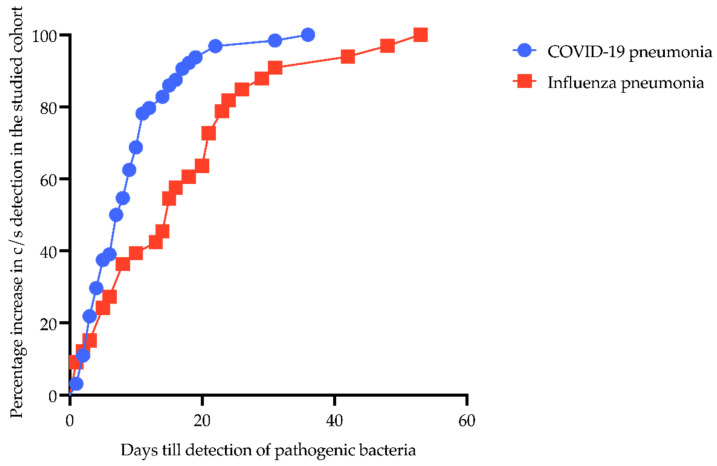
Percentage increase from hospital admission to detection of pathogenic bacteria in patients with COVID-19- and influenza-associated pneumonia. Y-axis shows percentage increase in c/s detection in the studied cohort; X-axis displays days till detection of pathogenic bacteria. Blue line with circles represents CP patients; red line with boxes shows IP patients.

The percentage increase in c/s detection plotted against days from hospital admission to detection of pathogenic bacteria in patients with CP and IP is shown in Figure 1. The time from admission to the detection of c/s in CP patients was significantly shorter than in IP patients (8.9 ± 6.9 days vs. 16.7 ± 13.2 days, *p* = 0.0028).

**Figure 2:**
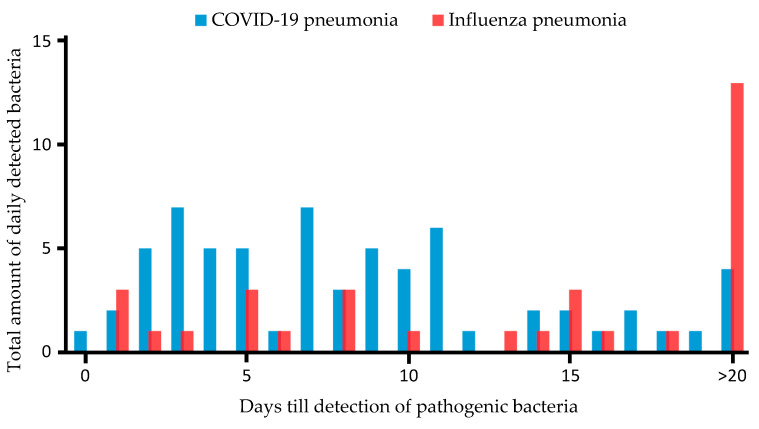
Time in days from hospital admission to detection of pathogenic bacteria in patients with COVID-19- and influenza-associated pneumonia. Y-axis shows total amount of pathogenic bacteria detected daily after ICU admission; X-axis displays days till detection of pathogenic bacteria. Blue bars represent CP patients; red bars show IP patients.

Figure 2 shows the absolute incidence of detection of pathogenic bacteria in patients with CP and IP, respectively.

The patients’ clinical characteristics are summarized in Table 2. Patients with IP were managed with higher positive end-expiratory pressure compared to patients with CP (16 ± 4 vs. 13 ± 4 cmH_2_O, *p* < 0.0001) and accumulated more carbon dioxide (69.6 ± 18.7 vs. 55.3 ± 18.6 mmHg, *p* < 0.0001) with consecutively impaired pH (7.2 ± 0.1 vs. 7.3 ± 0.1, *p* < 0.0001). Lactate levels at admission were significantly higher in patients with IP, and they showed a significantly higher incidence of acute myocardial dysfunction (56.6% vs. 25.4%, *p* < 0.0001), neurological events (38.2% vs. 15.8%, *p* = 0.0006) and multiorgan failure (94.7% vs. 69.3%, *p* < 0.0001). C-reactive protein (CRP) (223 ± 118 vs. 168 ± 99 mg/L, *p* = 0.0029) and procalcitonin (PCT) (58.5 ± 201.3 vs. 3.4 ± 11.3 µg/L, *p* < 0.0001) were significantly higher in the IP.

### 3.2. Bacterial Co-Infection or Superinfection

Bacteria causing co-infection in patients with viral pneumonia are summarized in Table 3. Overall, co-infection was detected in 15 (13.2%) of CP patients and 5 (6.6%) of IP patients (*p* = 0.2269). Multiple pathogenic bacteria were detected in 66.7% (10 of 15) of patients with CP and 40% (2 of 5) of patients with IP (*p* = 0.3473).

Bacteria causing superinfection in patients with viral pneumonia are summarized in Table 4. Superinfection was detected in 50 (43.9%) of the patients with CP and 28 (36.8%) of the patients with IP (*p* = 0.3687).

Considering the multiple detections of pathogenic bacteria in patients with superinfection, in patients with CP, we found multiple bacterial specimens in 50.0% of the patients (25 of 50) and with IP in 25% (7 of 28) (*p* = 0.0347).

Patients with CP were significantly more likely to show superinfection with Enterobacterales (74.0% vs. 46.4%, *p* = 0.0194), especially with Klebsiella pneumoniae (26% vs. 0%, *p* = 0.0019).

**Figure 3:**
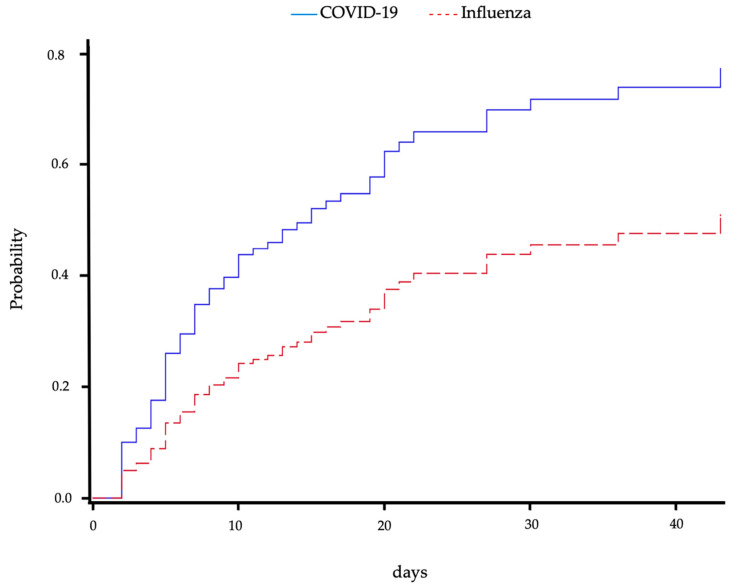
Cumulative incidence of superinfection over time for patients with COVID-19- or influenza-associated pneumonia. Mortality was regarded as a competing event. Blue solid line shows COVID-19-associated pneumonia patients, and red dashed line shows influenza-associated pneumonia patients.

In an analysis considering mortality as a competing event, the risk of superinfection in patients with CP was significantly higher compared to IP (sub-distribution hazard ratio (HR) 2.08 (95% CI 1.32–3.28), *p* = 0.0017) and is shown graphically in Figure 3. The cause-specific hazard ratio for superinfection was comparable with an about twofold higher risk in CP (HR 2.46 (95% CI 1.53–3.96), *p* = 0.0002). There was no difference in mortality in both groups (cause-specific HR 1.7 (95% CI 0.8–3.5), *p* = 0.1782 for CP vs. IP). These results were confirmed when the admission SOFA score was additionally considered (sub-distribution HR for superinfection with death as competing cause 1.79 (95% CI 1.03–3.10), *p* = 0.038; cause-specific HR for superinfection 2.12 (95% CI 1.196–3.764), *p* = 0.010). Mortality after superinfection did not differ between the groups (HR for CP compared to IP 1.38 (95% CI 0.34–1.56), *p* = 0.4115). This persisted when admission SOFA score, which was also not associated with mortality, was considered (HR for CP compared to IP, HR for admission SOFA 1.10 (95% CI 0.22–1.20), *p* = 0.2153).

### 3.3. Clinical Outcomes

We found no difference in mortality (35.1% vs. 38.6%, *p* = 0.7583) comparing patients with CP and IP (Table 5). Mortality in patients without c/s or with c/s also did not differ between the two groups (13.2% vs. 17.1%, *p* > 0.9999; 5.3% vs. 2.6%, *p* = 0.4795; and 16.7% vs. 18.4%, *p* = 0.8455) (Table 5).

Univariate and multivariate factors associated with mortality are presented in Table 6 and Table 7. Multivariable analysis identified cardiac arrest in patients with IP as an independent risk factor for mortality.

## 4. Discussion

In this retrospective observational, monocenter case-control study, we analyzed the incidence of c/s in a population of patients with either CP or IP in a tertiary referral center. We found the following:

(a) The incidence of c/s did not vary between patients with CP and IP;
(b) C/s did not contribute to mortality in our study population;
(c) Superinfection was primarily caused by Enterobacterales, especially *Klebsiella pneumoniae* in CP.

### 4.1. Incidence of Bacterial Co-Infection in COVID-19 and Influenza Patients

In a systematic review and meta-analysis by Lansbury et al. [4], a bacterial co-infection of 7% was reported for patients with CP, which increased to 14% when intensive care patients with CP were considered. A similar incidence was also found by Langford et al. [17]. Razazi et al. [18] found a significantly lower incidence of co-infections in patients with COVID-19-related ARDS compared to patients with non-SARS-CoV-2 viral ARDS. In contrast, Bergmann et al. [19] showed similar rates of early bacterial co-infection with CP and IP in ICU patients. Early bacterial co-infection was significantly associated with increased 30-day mortality in patients with CP in this study. Jorda et al. [20] showed comparable rates of community-acquired and hospital-acquired bacterial co-infections in hospitalized CP and IP patients. By contrast, other authors found an incidence of bacterial co-infections in patients with IP of 25–30% [21,22,23]. Synoptically, Lansbury et al. hypothesized that bacterial co-infection is less prevalent in patients with CP than in those with IP [4]. However, bacterial co-infections were associated with significantly increased morbidity and mortality in IP patients [8,21,22,23]. In our study, we found a similar incidence of bacterial co-infections, with 13.2% for patients with CP and 6.6% for patients with IP.

### 4.2. Incidence of Bacterial Superinfection in COVID-19 and Influenza Patients

Regarding bacterial superinfections, an incidence of 4.7% to 22% is reported in patients with CP and 4% to 53% in patients with IP [5,6,24,25,26]. Except for Rozé et al. [27], no other study showed similar incidences of bacterial superinfections in patients with CP compared to our study.

This may be due to the fact that the present study considered only patients receiving therapy in ICU in a tertiary referral center treating predominantly mechanical ventilated patients with moderate to severe respiratory failure. This is also reflected by the comparatively high SOFA score and severity of the disease of the patients rendering them susceptible to bacterial superinfection (Table 1). In comparison, studies investigating patients with CP typically showed distinctly lower SOFA scores [28,29,30]. On the other hand, for patients with co-infection with IP requiring ECMO, Rozencwajg et al. [8] showed similar high SOFA scores for patients with additional co-infection. In a study by Falcone et al. [25], including 315 patients, the median time from hospital admission to the diagnosis of bacterial superinfection in patients with CP was reported to be 19 days. Rothberg et al. [31] reported bacterial superinfection between days 4 and 14 in IP patients. In our cohort, superinfections in CP were detected markedly earlier, with a mean of 8.9 days. In IP patients, bacterial superinfections were detected considerably later, at 16.7 days. In comparison, pathogenic bacteria were significantly earlier detected in CP than in IP in the studied cohort (Figure 1).

In patients with ARDS caused by COVID-19 in contrast to other viral pathogens, Razazi et al. [18] compared the incidence of ventilator-associated pneumonia and invasive aspergillosis with a competing risk analysis adjusting for mortality and ventilator weaning. They found a significantly higher probability of ventilator-associated pneumonia in COVID-19-related ARDS. The probability of successful ventilator weaning in CP was significantly lower when ventilator-associated pneumonia and mortality were considered competing events.

We also regarded the risk of superinfection over time, considering mortality as a competing event as well as discharge as censored, and found a significantly higher probability of superinfection for patients with CP compared to patients with IP.

### 4.3. Characteristics of Co-Infection or Superinfection in Patients COVID-19 or Influenza Patients

According to the literature, the most frequently reported bacteria causing c/s in patients with IP were *Staphylococcus aureus*, *Streptococcus pneumoniae*, and *Haemophilus influenzae* [8,10,21,22,32]. In patients with CP and c/s, different authors showed a divergent spectrum of bacteria, namely *Mycoplasma pneumoniae*, *Pseudomonas aeruginosa*, *Haemophilus influenzae, Staphylococcus aureus* and *Klebsiella pneumoniae* [4,15,27,33]. In contrast to IP, the pathophysiological mechanisms of superinfection in CP are not fully understood. Discussed factors by which viruses predispose to secondary bacterial infection are damage to airway epithelium, diminished ciliary beat frequency, and dysregulation of the immune response [34]. The possible mechanistic differences could explain changes in the detected bacterial spectrum. To date, however, no study has examined the differences between CP and IP in the context of c/s. In our retrospective data analysis of prospectively collected data, we found significantly more Enterobacterales, particularly *Klebsiella pneumonia*, in patients with CP compared to patients with IP (Table 3). This result is consistent with the currently available literature [4,15,27]. The frequent detection of superinfections with Gram-negative bacteria in CP may not be related to specific mechanisms predisposing patients with CP to Gram-negative pathogens but to events like microaspiration causing ventilator-associated pneumonia.

### 4.4. Outcome of Bacterial Co-Infection in COVID-19 and Influenza Patients

In their multicenter retrospective study including 13,781 patients, Patton et al. [3] demonstrated that bacterial co-infection in patients with CP is associated with increased ICU admission, mechanical ventilation, and in-hospital mortality compared with CP without co-infection. Garcia-Vidal et al. [5] also showed that patients with a CP with bacterial co-infection were frequently admitted to an ICU and had higher mortality.

Rouzé et al. [35] demonstrated a significantly lower incidence of bacterial co-infections (9.7 vs. 33.6%) within 48h after intubation in patients with CP compared to IP. Nonetheless, they found no effects of co-infection on mortality.

In a multicenter study including 1349 patients, Delhommeau et al. [36] found significantly higher mortality at day 60 in patients with CP compared to IP. In accordance with our data, the mortality risk did not increase due to c/s.

In a multivariate analysis including 77 patients, Rozencwajg et al. [8] showed that a bacterial co-infection in patients with IP supported by veno-venous ECMO is an independent predictor of hospital mortality. A pooled analysis of the odds ratios for death, including four studies with 733 patients [28,29,37,38] by Lansbury et al. [4], also showed higher mortality for patients with CP and bacterial co-infection than without.

However, bacterial co-infection was not identified as an independent risk factor for mortality in our study (Table 5, Table 6 and Table 7). Also, the type of viral pneumonia showed no significant difference regarding mortality in the studied cohort.

### 4.5. Outcome of Bacterial Superinfection in COVID-19 and Influenza Patients

In a study including 989 patients, Garcia-Vidal et al. [5] showed that patients with CP and superinfection had a significantly prolonged length of stay in the hospital and higher mortality. Falcone et al. [25] and Yoon et al. [39] also showed a significantly prolonged length of stay for patients with CP and superinfection but no significant association between superinfection and mortality in these patients. Wallemacq et al. [40] demonstrated an increased mortality in CP patients compared to IP patients independent of bacterial superinfection, which, according to the authors, suggests that the increased mortality rate in CP is not related to secondary complications but is likely due to the severity of the disease. Also, Wicky et al. [41], in their multicentric observational study, found a cumulative incidence rate of superinfection of 39.5%. However, this also had no effect on mortality. In contrast, in their multicenter study including 73,945 patients, Vacheron et al. [42] found an increase of 9% in mortality attributable to ventilator-associated pneumonia in CP. MacIntyre et al. [21] summarized in their systematic review, including 75 studies, that secondary bacterial infection was an important complication during the 2009 influenza pandemic associated with morbidity and mortality.

Although patients with CP in the studied cohort had a significantly higher probability of superinfection over time, neither the type of viral pneumonia nor bacterial superinfection was identified as an independent risk factor for hospital mortality in our study (Table 5, Table 6 and Table 7). The high severity of illness at admission to the ICU in our cohort might be a possible explanation for this finding [8,24,25,28,29].

## 5. Limitations

Our study has several limitations. First, we only analyzed patients treated in a tertiary referral center specializing in the management of patients with severe respiratory dysfunction. This is a clear selection bias represented by the high disease severity in our study population. Second, as the incidence of IP was extremely low during the pandemic, patients with IP respectively CP were not treated at the same time. So we cannot exclude systematic changes in the general management of patients with severe pneumonia. Third, there are intrinsic difficulties differentiating c/s in patients with viral pneumonia. Several authors have already criticized the inconsistent definition of c/s. This leads to an inadequate determination of the prevalence and the associated morbidity [3,15,17]. However, the definitions of c/s used in our analysis are equivalent to recent publications [4,5,6,8,14,15,16]. Additionally, in this study, each microbiological isolate was reviewed by an experienced senior ICU consultant to determine the clinical significance; bacteria identified but not warranting specific therapy were defined as commensal and nonsignificant. Last, due to the retrospective design of this study, we could not perform a preceding power analysis.

## 6. Conclusions

C/s are common in critically ill patients with CP and IP but are not an independent predictor of mortality in a study cohort with a high disease severity. The overall incidence of c/s did not differ between CP and IP patients; however, Gram-negative pathogens were more frequently detected in patients with CP. We found a significantly higher probability of superinfection for patients with CP compared to patients with IP.

## Figures and Tables

**Table 1:** Anthropometric characteristics of patients included in this study.

Characteristic	COVID-19 (*n* = 114)	Influenza (*n* = 76)	*p*-Value
Sex (male/female)	73/41	46/30	0.65
Age (years)	61 ± 16	49 ± 13	**<0.0001**
Height (cm)	172 ± 9	174 ± 9	0.30
Weight (kg)	86.5 ± 20.8	88 ± 18.1	0.83
BMI (kg/m^2^)	29.2 ± 6.6	30 ± 5.7	0.42
Pre-existing conditions			
Chronic respiratory disease	13 (11.4%)	11 (14.5%)	0.66
Chronic heart disease	40 (35.1%)	26 (34.2%)	>0.99
Chronic renal disease	10 (8.8%)	3 (4%)	0.25
Chronic liver disease	0 (0%)	2 (2.6%)	0.16
Diabetes mellitus	23 (20.2%)	29 (38.2%)	**0.0080**
Immunocompromised	18 (6.1%)	27 (35.5%)	**<0.0028**
ICU length of stay (days)	18 ± 16	21 ± 16	0.07
Hospital length of stay (days)	23 ± 16	22 ± 16	0.69
Mechanical ventilation at admission	89 (78.1%)	75 (98.7%)	**<0.0001**
Mechanical ventilation duration (days)	17± 16	20 ± 15	**0.0377**
Days till bacteria detection (days)	9 ± 7	17 ± 13	**0.0028**
No ARDS	26 (22.8%)	3 (4.0%)	**0.0003**
Mild ARDS	7 (6.1%)	10 (13.2%)	0.1211
Moderate ARDS	36 (31.6%)	30 (39.5%)	0.2796
Severe ARDS	45 (39.5%)	33 (43.4%)	0.6522
SAPS II score at admission	51.3 ± 19.7	42.3 ± 12.7	**0.0012**
SOFA score at admission	9.4 ± 3.9	13.4 ± 2.3	**<0.0001**
ECMO	28 (24.6%)	37 (48.7%)	**0.0010**
ECMO duration (days)	20 ± 12	18 ± 13	0.47

Comparison of patients with CP and IP. Values are numbers (proportions) or means ± standard deviation for 114 patients with CP and 76 patients with IP. Data were analyzed with Student’s *t*-test, Mann–Whitney *U* test, or Fisher’s exact test as appropriate (*p* < 0.05). Bold letters represent statistically significant differences. BMI = body mass index; ICU = intensive care unit; SAPS II = simplified acute physiology score II; SOFA = sequential organ failure assessment; ECMO = extracorporeal membrane oxygenation.

**Table 2:** Clinical characteristics of patients included in this study.

Characteristic	COVID-19 (*n* = 114)	Influenza (*n* = 76)	*p*-Value
Respiratory rate at admission (1/min)	24 ± 5	19 ± 7	**<0.0001**
PEEP at admission (cmH_2_O)	13 ± 4	16 ± 4	**<0.0001**
Driving pressure at admission	17 ± 6	17 ± 6	0.95
PaO_2_/F_i_O_2_ at admission (mmHg)	121 ± 63	134 ± 74	0.30
paCO_2_ at admission (mmHg)	55 ± 19	70 ± 19	**<0.0001**
pH at admission	7.3 ± 0.1	7.2 ± 0.1	**<0.0001**
Lactate at admission (mmol/L)	2.1 ± 2.2	3.0 ± 3.4	**0.0143**
Renal replacement therapy	59 (51.8%)	41 (54%)	0.88
Cardiogenic shock	29 (25.4%)	43 (56.6%)	**<0.0001**
Neurological event	18 (15.8%)	29 (38.2%)	**0.0006**
Multiorgan failure	79 (69.3%)	72 (94.7%)	**<0.0001**
Leukocytes at admission (1/nL)	13 ± 12	12 ± 9	0.57
CRP at admission (mg/L)	168 ± 99	223 ± 118	**0.0029**
PCT at admission (µg/L)	3.4 ± 11.3	58.5 ± 201.3	**<0.0001**

Comparison of patients with CP and IP. Values are numbers (proportions) or means ± standard deviation for 114 patients with CP and 76 patients with IP. Data were analyzed with Student’s-*t*-test, Mann–Whitney *U* test, or Fisher’s exact test as appropriate (*p* < 0.05). Bold letters represent statistically significant differences. PEEP = positive end-expiratory pressure; PaO_2_/F_i_O_2_ = partial oxygen pressure in arterial blood/fraction of inspired oxygen ratio; paCO_2_ = partial carbon dioxide pressure in arterial blood; CRP = C-reactive protein; PCT = procalcitonin.

**Table 3:** Bacterial co-infection in patients with proven COVID-19- or influenza-associated pneumonia.

	COVID-19 (*n* = 114)	Influenza (*n* = 76)	*p*-Value
Bacterial co-infection	15 (13.2%)	5 (6.6%)	0.2269
Multiple pathogenic bacteria	10 (66.7%)	2 (40.0%)	0.3473
Gram-positive	13 (86.7%)	5 (100%)	0.3194
*Staphylococcus aureus*	13 (86.7%)	5 (100%)	0.3194
*Streptococcus pneumoniae*	1 (6.7%)	0 (0%)	>0.9999
*Enterococcus* spp.	1 (6.7%)	0 (0%)	>0.9999
Gram-negative	9 (60%)	2 (40.0%)	0.2045
*Pseudomonas aeruginosa*	2 (13.3%)	0 (0%)	0.5175
*Burkholderia cepacia*	0 (0%)	0 (0%)	>0.9999
*Proteus mirabilis*	2 (13.3%)	0 (0%)	0.5175
*Proteus vulgaris*	1 (6.7%)	0 (0%)	>0.9999
*Morganella morganii*	1 (6.7%)	0 (0%)	>0.9999
*Haemophilus influenzae*	0 (0%)	0 (0%)	>0.9999
*Stenotrophomonas maltophilia*	2 (13.3%)	0 (0%)	0.5175
*Acinetobacter baumanii*	0 (0%)	1 (20.0%)	0.4000
Enterobacterales	8 (53.3%)	2 (40.0%)	0.3202
*Citrobacter freundii*	0 (0%)	0 (0%)	>0.9999
*Citrobacter koseri*	2 (13.3%)	0 (0%)	0.5175
*Enterobacter cloacae*	3 (20%)	1 (20.0%)	0.6510
*Escherichia coli*	2 (13.3%)	0 (0%)	0.5175
*Klebsiella oxytoca*	1 (6.7%)	1 (20.0%)	>0.9999
*Klebsiella aerogenes*	0 (0%)	0 (0%)	>0.9999
*Klebsiella pneumoniae*	4 (26.7%)	0 (0%)	0.1512
*Serratia marcescens*	2 (13.3%)	0 (0%)	0.5175

Data show all detected pathogenic bacteria in tracheal aspirate or bronchoalveolar lavage in 114 patients with CP and 76 patients with IP. If multiple pathogenic bacteria were detected in a patient, this was considered as one co-infection. However, the different bacteria were also considered in the analysis. Values are numbers (proportions). Data were analyzed with Fisher’s exact test (*p* < 0.05); spp. = species pluralis.

**Table 4:** Bacterial superinfection in patients with proven COVID-19- or influenza-associated pneumonia.

	COVID-19 (*n* = 114)	Influenza (*n* = 76)	*p*-Value
Bacterial superinfection	50 (43.9%)	28 (36.8%)	0.3687
Multiple pathogenic bacteria	25 (50.0%)	7 (25.0%)	**0.0347**
Gram-positive	25 (50.0%)	16 (57.1%)	>0.9999
*Staphylococcus aureus*	16 (32.0%)	7 (25.0%)	0.3702
*Streptococcus pneumoniae*	1 (2.0%)	0 (0%)	>0.9999
*Enterococcus* spp.	8 (16.0%)	0 (0%)	**0.0226**
Gram-negative	44 (88.0%)	20 (71.4%)	0.0868
*Pseudomonas aeruginosa*	8 (16.0%)	7 (25.0%)	0.5938
*Burkholderia cepacia*	1 (2.0%)	1 (3.6%)	>0.9999
*Proteus mirabilis*	1 (2.0%)	2 (7.1%)	0.5649
*Proteus vulgaris*	1 (2.0%)	0 (0%)	>0.9999
*Morganella morganii*	1 (2.0%)	0 (0%)	>0.9999
*Haemophilus influenzae*	3 (6.0%)	0 (0%)	0.2762
*Stenotrophomonas maltophilia*	1 (2.0%)	1 (3.6%)	>0.9999
*Acinetobacter baumanii*	1 (2.0%)	0 (0%)	>0.9999
Enterobacterales	37 (74.0%)	13 (46.4%)	**0.0194**
*Citrobacter freundii*	0 (0%)	1 (3.6%)	0.4000
*Citrobacter koseri*	9 (18.0%)	2 (7.1%)	0.2045
*Enterobacter cloacae*	6 (12.0%)	3 (10.7%)	0.7433
*Escherichia coli*	14 (28.0%)	5 (17.9%)	0.2270
*Klebsiella oxytoca*	4 (8.0%)	1 (3.6%)	0.6498
*Klebsiella aerogenes*	4 (8.0%)	3 (10.7%)	>0.9999
*Klebsiella pneumoniae*	13 (26.0%)	0 (0%)	**0.0019**
*Serratia marcescens*	3 (6.0%)	0 (0%)	0.2762

Data show all detected pathogenic bacteria in tracheal aspirate or bronchoalveolar lavage in 114 patients with CP and 76 patients with IP. If multiple pathogenic bacteria were detected in a patient, this was considered as one superinfection. However, the different bacteria were also considered in the analysis. Values are numbers (proportions). Data were analyzed with Fisher’s exact test (*p* < 0.05). Bold letters represent statistically significant differences; spp. = species pluralis.

**Table 5:** Outcomes of patients with COVID-19- and influenza-associated pneumonia, as well as infection and concomitant bacterial co-infection or superinfection.

	COVID-19 (*n* = 114)	Influenza (*n* = 76)	*p*-Value
Mortality (total)	40 (35.1%)	29 (38.6%)	0.7583
Mortality without co-infection or superinfection	15 (13.2%)	13 (17.1%)	>0.9999
Mortality + bacterial co-infection	6 (5.3%)	2 (2.6%)	0.4795
Mortality + bacterial superinfection	19 (16.7%)	14 (18.4%)	0.8455

Data are presented as absolute values and percentages. Data were analyzed with Fisher’s exact test (*p* < 0.05).

**Table 6:** Univariable and multivariable analysis of factors associated with mortality for patients with COVID-19-associated pneumonia.

	Univariate Analysis		Multivariable Analysis	
Parameter	Odds Ratio (95%CI)	*p*-Value	Odds Ratio (95%CI)	*p*-Value
Female sex	0.9 (0.4–2.1)	0.8746		
Age ≥ 65 years	3.9 (1.7–8.9)	**0.0011**	2.3 (0.7–7.6)	0.1462
BMI ≥ 35 kg/m^2^	1.3 (0.4–3.8)	0.6693		
Chronic respiratory disease	0.8 (0.2–2.7)	0.7293		
Chronic heart disease	3.2 (1.4–7.3)	**0.0049**	1.5 (0.5–4.6)	0.4561
Chronic renal disease	1.3 (0.3–4.7)	0.7337		
Diabetes mellitus	1.2 (0.5–3.2)	0.6497		
Immunocompromised	1.2 (0.4–3.4)	0.7130		
SAPS II at ICU admission ≥ 42	4.6 (1.7–14.9)	**<** **0.0044**	1.6 (0.5–6.4)	0.4571
SOFA at ICU admission ≥ 12	4.0 (1.6–10.6)	**<** **0.0036**	1.5 (0.4–5.7)	0.5511
ECMO requirement	2.3 (1.0–5.6)	0.7293		
Driving Pressure > 14 cmH_2_O	2.4 (1.1–5.5)	**0.0267**	1.0 (0.3–3.5)	0.9697
Lactate ≥ 2 mmol/L at admission	3.5 (1.5–8.4)	**0.0049**	1.8 (0.5–6.3)	0.3609
Renal replacement therapy	3.2 (1.5–7.5)	**0.0049**	1.2 (0.3–4.4)	0.7796
Cardiogenic shock	3.1 (1.3–7.6)	**0.0103**	2.1 (0.6–7.2)	0.2194
Multiorgan failure	6.5 (2.4–19.9)	**0.0012**	2.8 (0.6–16.5)	0.3651
Cardiac arrest	/	/		
Co-infection	1.3 (0.4–3.8)	0.6693		
Superinfection	1.3 (0.6–2.7)	0.5650		
Gram-positive	1.2 (0.5–2.6)	0.6455		
Gram-negative	1.2 (0.6–2.7)	0.5810		

Univariable analysis of factors associated with mortality was performed using logistic regression. Multivariable analysis was used to further evaluate factors significantly associated with mortality in the univariable analysis. Bold letters represent statistically significant differences. CI = confidence interval; BMI = body mass index; SAPS II = simplified acute physiology score II; ICU = intensive care unit; SOFA = sequential organ failure assessment; ECMO = extracorporeal membrane oxygenation.

**Table 7:** Univariable and multivariable analysis of factors associated with mortality for patients with influenza-associated pneumonia.

	Univariate Analysis		Multivariable Analysis	
Parameter	Odds Ratio (95%CI)	*p*-Value	Odds Ratio (95%CI)	*p*-Value
Female sex	2.3 (0.9–6.0)	0.0888		
Age ≥ 65 years	2.2 (0.5–9.8)	0.2612		
BMI ≥ 35 kg/m^2^	0.6 (0.1–3.5)	0.5709		
Chronic respiratory disease	0.3 (0.04–1.3)	0.1570		
Chronic heart disease	1.7 (0.6–4.4)	0.3026		
Chronic renal disease	3.4 (0.3–75.3)	0.3261		
Diabetes mellitus	1.6 (0.6–4.1)	0.3484		
Immunocompromised	3.1 (1.2–8.5)	**0.0227**	1.5 (0.1–2392)	0.9388
SAPS II at ICU admission ≥ 42	3.9 (1.5–10.5)	**0.0052**	2.5 (0.2–37.3)	0.4548
SOFA at ICU admission ≥ 12	12.3 (2.2–229.0)	**0.0189**	1.3 (0.1–237.8)	0.8989
ECMO requirement	5.1 (1.9–14.7)	**0.0017**	5.8 (0.6–173.2)	0.1867
Driving Pressure > 14 cmH_2_O	1.0 (0.4–2.9)	0.9361		
Lactate ≥ 2 mmol/L at admission	3.5 (1.3–9.4)	**0.0144**	1.8 (0.2–22.4)	0.6177
Renal replacement therapy	1.4 (0.5–3.5)	0.5213		
Cardiogenic shock	5.2 (1.9–16.2)	**0.0026**	3.0 (0.3–48.7)	0.3814
Multiorgan failure	/	**/**		
Cardiac arrest	145.1 (30.4–1158)	**<0.0001**	217.8 (26.9–6849)	**<0.0001**
Co-infection	1.0 (0.1–7.0)	0.9301		
Superinfection	2.2 (0.8–5.8)	0.1074		
Gram-positive	2.3 (0.9–6.0)	0.0868		
Gram-negative	1.2 (0.4–3.2)	0.7528		

Univariable analysis of factors associated with mortality was performed using logistic regression. Multivariable analysis was used to further evaluate factors significantly associated with mortality in the univariable analysis. Bold letters represent statistically significant differences. CI = confidence interval; BMI = body mass index; SAPS II = simplified acute physiology score II; ICU = intensive care unit; SOFA = sequential organ failure assessment; ECMO = extracorporeal membrane oxygenation.

## Data Availability

The datasets analyzed during this study are available from the corresponding author on reasonable request.
